# Qualitative perspectives of isolation experiences due to COVID-19 from a group of bioethicists in training performing interdisciplinary healthcare activities. Medellin, Colombia. September 2020

**DOI:** 10.47626/1679-4435-2022-874

**Published:** 2023-08-08

**Authors:** John Camilo Garcia-Uribe, María Osley Garzón-Duque, Mónica María Massaro-Ceballos, Luz Adriana Espinal-Espinal, Irma Del Carmen Canastero-Montoya, Cristina Posada-Giraldo, Carlos Alberto Gallo-Orjuela, María Margarita Villa-García, María Isabel Erazo-Dilson, Sol Beatriz Castro-Arango

**Affiliations:** 1 Universidad CES – Facultad de Medicina, Medellín, Antioquia, Colombia; 2 Corporación Universitaria Remington. Facultad de Ciencias de la Salud – Grupo de Investigación Salud Familiar y Comunitaria; 3 Instituto Neurológico de Colombia, Epidemiologia, Medellín, Antioquia, Colombia; 4 Instituto Neorológico de Colombia, Dirección Médica, Medellín, Antioquia, Colombia; 5 Clínica Las Américas, Médica de apoyo cuidados paliativos, Medellín, Antioquia, Colombia; 6 IPS Universitaria, Dolor y Cuidados paliativos, Medellín, Antioquia, Colombia; 7 Universidad Pontificia Bolivariana, Facultad de Medicina, Medellín, Antioquia, Colombia; 8 Samein, Psiquiatria, Medellín, Antioquia, Colombia; 9 Universidad CES, CECIF, Centro de Ciencia e Investigación Farmacéutica, Medellín, Antioquia, Colombia

**Keywords:** Patient isolation, mental health, health personnel, COVID-19, Aislamiento de pacientes, salud mental, personal de salud, COVID-19

## Abstract

**Introduction:**

In Colombia, there is still little information on how health care personnel have lived and coped with isolation due to COVID-19.

**Objectives:**

To explore the experiences related to the isolation of health professionals performing interdisciplinary care activities from March to September 2020, in Medellín, Colombia.

**Methods:**

Qualitative, exploratory, with a group of bioethicists in training. Data collected through the focus group, after obtaining the consent and approval of the Institutional Ethics Committee. Open and axial coding was performed. Texts are presented in prose, information was triangulated, and results were validated with the participants.

**Results:**

Work increased and staff decreased, with high staff turnover, redistribution and reassignment of loads and roles, facilitating physical and emotional overload. Study participants considered that teleworking facilitated their work, although more work was done. They lived in double isolation, had losses, and took work and family overloads. For fear of infecting and being infected, they separated from their loved ones, “this is an absolutely lonely disease, if people does not die from COVID, sadness and loneliness kills them.” It affected “the recovery process, specifically, of psychiatric patients was prolonged, worsening their condition.” They live in the present, and prioritize what is most important, because “being healthy and having those you love is the best wealth”.

**Conclusion:**

Isolation increased workload, with reassignment of roles, affecting health care. For fear of becoming infected and infecting, study participants lived a double isolation, with anguish and uncertainty, which is why now they prioritize the most important health and love.

## Abstract (pt)

**Introducción:**

En Colombia, todavía hay pocas informaciones sobre cómo los profesionales sanitarios han vivido y afrontado el aislamiento por la COVID-19.

**Objetivos:**

Explorar las experiencias relacionadas al aislamiento de profesionales sanitarios con actividad asistencial interdisciplinar, entre marzo y septiembre de 2020, en Medellín, Colombia.

**Métodos:**

Se condujo una investigación cualitativa y exploratoria con un grupo de bioeticistas en formación. Datos recolectados a través de un grupo focal, después de obtener el consentimiento y la aprobación del Comité de Ética Institucional. Se realizó codificación abierta y axial. Los textos se presentan en prosa, se hizo triangulación de la información, y se validaron los resultados con los participantes.

**Resultados:**

El trabajo aumentó y el personal disminuyó, con alta rotación de personal, reasignación y redistribución de cargas y roles, facilitando la sobrecarga física y emocional. Los participantes consideraron que el teletrabajo facilitó su trabajo, aunque se ha hecho más trabajo. Además, hubo un doble aislamiento, pérdidas y sobrecargas de trabajo y familiares. Por miedo a infectar e infectarse, se separaron de sus seres queridos, “esta es una enfermedad absolutamente solitaria, si uno no muere de la COVID, lo matan la tristeza y la soledad.” La pandemia también afectó el proceso de recuperación, específicamente de los pacientes psiquiátricos, que se alargó, empeorando su condición. Los profesionales viven el presente y priorizan lo más importante: “tener salud y a los que amas es la mejor riqueza”.

**Conclusión:**

El aislamiento aumentó la carga de trabajo, con reasignación de roles, afectando la atención sanitaria. Por miedo a infectarse e infectar, los participantes del estudio vivieron un doble aislamiento, con angustia e incertidumbre, y ahora priorizan la salud y el amor.

## INTRODUCTION

Hospital isolation is intended to prevent the transmission of micro-organisms to patients, hospital staff, and visitors, through the interruption of the epidemiological chain that facilitates their transmission, where factors related to the host and the pathogen agent are more difficult to control, activities are directed to transmission mechanisms,^1^ and physical and spatial barriers are imposed between source of infection (colonized or infected patient) and other patients, health personnel, and visitors.^1^ However, there is still scarce comprehensive information about this isolation at the time of COVID-19 pandemic from the health personnel perspective.

Although the Centers for Disease Control and Prevention (CDC) released the first publication with evidence-based guidelines for the implementation of hospital isolation units in 1970,^1^ isolation as a method to segregate patients have been practiced since ancient times, being one of the first public health measures, as shown in the Bible,^2^ where the following words appear: “When a man shall have in the skin of his flesh a rising, a scab, or bright spot, [...] it is a plague of leprosy and this man shall be pronounced unclean [...] all the days wherein the plague shall be in him he shall be defiled [... ] he shall dwell alone; without the camp shall his habitation be, every person or object that touches him shall be considered unclean.”^2^

Subsequently, during the Black Death epidemic in Europe, it was stated that every person with plague should be isolated in camps outside the town to die or recover.^3^ During the same period, the “trentino rule” (30-day isolation) was established for individuals coming from areas where the plague was endemic. Subsequently, the time of isolation was extended to 40 days, which is why it was named quarantino,^3^ hence quarantine. Currently, quarantine applies to people who have been exposed to an infectious disease and do not have any symptoms yet^4^; however, isolation implies the imposition of physical and spatial barriers to prevent the transmission of pathogen agents.^4^ These measures are considered additional ones, because they are added to standard measures to prevent and control infections (applied to all patients).

Etymologically, isolate is derived from the word island”^5^ and means separating or setting someone apart, placing them on an island, metaphorically speaking. Hospital comes from the Latin *hospes,* deriving in the words host and hostile, means the place where sick people are assisted.^6^ Hospital isolation leads to a double patient’s segregation, separating them from healthy people and, in the hospital, from patients with other non-infectious diseases. Currently, in COVID-19 times, this separation becomes especially significant; the pandemic obliged people to dramatically change their lifestyle, in an effort to flatten the epidemic curve,^7^ allowing for the health systems to gain time to respond to an emerging and highly contagious virus. These dramatic lifestyle changes, the presence of an invisible pathogen agent, uncertainty, fake news,^8^ exacerbation of socioeconomic problems,^9^ precariousness of some health systems, surrounding fear, belligerent language, and the shift towards a utilitarian ethics^10^ have had a significant influence on the doctor-patient relationship.

For the aforementioned reasons, the present study aimed to explore the experiences of a group of health care professionals performing interdisciplinary care activities, related with isolation in pandemic times, especially concerning their working life and decisionmaking experiences from March to September 2020.

## METHODS

An exploratory study was conducted using tools for ethnographic qualitative research, in order to investigate experiences related to patient isolation due to COVID-19 in a group of bioethicists in training from health institutions in Medellín, Colombia. The techniques of observation and group interview (focus group, FG) were used, which have already been used in similar investigations and groups works.^11,12^

### VALIDITY AND QUALITY CRITERIA

Previous acquaintance among participants facilitated an environment of confidence and dialogue, allowing for them to agree with regard to the study object. Under the premise of respect, the group of participants reached a consensus, stating the confidentiality of the provided information. The FG was transcribed textually, information was triangulated among investigators, results were validated with the participants, participants’ statements are cited in data interpretation and analysis, and the methodological route is described in detail.

### PARTICIPANTS

The study included nine Bioethics masters’ students, who had shared their training and life experiences for 1.5 years in a university in the city of Medellín, Colombia. The academic activity of the FG was planned, coordinated, and supervised with the assistance of the professor, leader of the course on qualitative research fundamentals. The study team included a moderator, an note-taker, a logistic coordinator, and thematic experts: two specialist doctors (a palliativist and a psychiatrist), a general physician, and a professional nurse, who worked in the front line of COVID-19 care; two specialist doctors who worked in the management of health care decision-making (hospital epidemiology and medical management) of health institutions providing care to patients with COVID in Valle de Aburrá; and a pharmaceutical chemist, who intervenes in the biomedical supply production chain.

### TECHNIQUES

The techniques used and the assigned roles were previously agreed. The FG took place through a technology assisted face-to-face meeting via zoom. During the entire session, connectivity was checked, and it was ensured that participants were visualized in the screenshot so as to register their non-verbal language.

### OBSERVATION AND FIELD DIARY

A note-taker recorded verbal and non-verbal languages, consensuses and disagreements between participants, and information about the dynamic of the group and the environment where they were placed. Both logistic assistant and moderator took notes of statements in interventions. Photographs were obtained on three occasions, and the session was recorded, after patients’ verbal and written consent.

### GROUP INTERVIEW

After the theme was introduced, participants asked five questions (working life during the pandemic, experiences with patients isolated due to COVID-19, influence on personal, working, family, and social life, coping strategies, and how bioethics can improve the approach of isolated patients), and then the session was closed.

### ANALYSIS OF INFORMATION

Analytical tools of the grounded theory were employed,^13^ proceeding with the textual transcription of the group interview and the notes in order to categorize information, according to the thematic areas pre-established by the questions, performing a constant comparative analysis, grouping and ungrouping data, through open coding, before recategorization of themes and subthemes, according to the order derived from initial analysis. Subsequently, maps were created, and then relevant statements for each question were selected. Furthermore, notes on verbal and non-verbal language were analyzed, as well as discourses obtained to refine the selection of participant discourses, which would accompany the interpretation and analysis of themes and subthemes, keeping in mind the identified agreements and disagreements. Finally, results were shared among investigators to make adjustments and to complement the contents on each theme pre-established for the study.

### ETHICAL ASPECTS OF THE STUDY

Participants’ privacy and confidentiality were guaranteed, as well as the accuracy of information from the perspective of each participant, and informed consent was obtained before the FG was conducted, as well authorization for recording, photograph taking, and scientific publication. There were no conflicts of interest to declare. This study was approved by the Research Ethics Committee of Universidad CES (extended approval protocol #58).

## RESULTS

The main findings of the pre-established categories and themes will be present, as well as emergent ones. Firstly, this study addresses the implications of the changes in the labor logics of study participants, which generate greater workload and burnout, since they involved providing a response to an unexpected and chaotic situation, generating several actions and reactions, with the loss of boundaries between work and life. Furthermore, we present the main contribution with regard to participants’ feelings and values, which were permeated by the fear of contaminate and being contaminated, of the disease, of multiple isolation (patients, family, and society), in addition to the feeling of uncertainty, anguish, and powerlessness and to experiencing loneliness from the situation the participants should face, in which they experienced changes, thoughts of uncertainty over the future and about death. These conditions were intensified by lack of knowledge, infodemic, and lack of preparation. Conversely, loneliness resulting from isolation leads to emotional destabilization and distress, due to separation from their families, despite the use of communication technologies.

**Figure 1:**
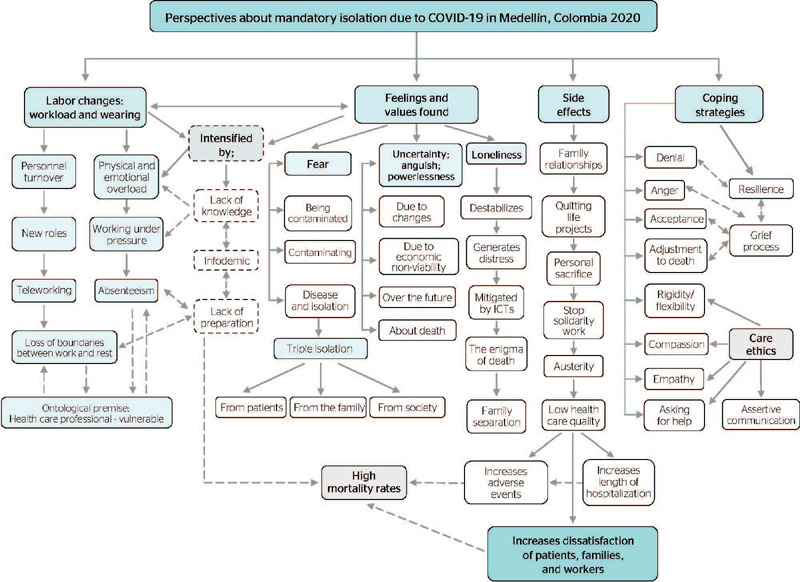
Qualitative perspectives of isolation experiences due to COVID-19 in a group of bioethicists in training performing interdisciplinary care activities. Medellín, Colombia. September 2020. ITCS = Information Technologies for Health.

One relevant theme was the side effects of mandatory isolation, which affected participants’ family relationships and obliged them to abandon their life projects and make sacrifices at the individual level. They also felt less solidarity, and, due to austerity that should be adopted by their institutions, they felt a decrease in the quality of care provided, with an increase in hospital length of stay and in the number of adverse events. Finally, we present the coping strategies use, which included denial, anger, acceptance, adjustment to death, learning to deal with grief, and resilience, without neglecting care ethics, seeing and experiencing compassion, empathy, going from rigidity to flexibility, and advancing in assertive communication (Figure 1).

### WORK AND WORKLOAD

The pandemic affected work dynamic, work distribution, and professionals’ roles, “there was absenteeism due to disabilities” MGF_IC1. “The first patient diagnosed with COVID contaminated many others, put us in a tight spot” and “we had to close beds in several services because there were no personnel to care for the patients, it was a very hard time” GF_JCG1. Personnel turnover affected work situation also, increasing role reassignment and dissatisfaction. “We had to implement a system of temporary workers [...] that caused dissatisfaction, because they had experience in other areas and now had to support COVID areas in the ICU [intensive care unit] and SCU [special care unit]. Furthermore, “Staff reassignment was more complex, due to their comorbidities and restrictions” GF_JCG1, they should take different roles with few hands, “for example, to prepare cadavers; one an assistant and me to pack them, it think this was the hardest experience so far, I’ll never forget it” MGF-2PC.

Professionals experienced physical and emotional overload, “the work increased, the personal was reduced, we left later and started earlier, [...] often without enough rest and with patients dying every day, aged both 80 years old and 15” MGF_CP1, and this affected not only the care staff, because; “in pharmaceutical practice, workload tripled also, it was chaotic [...], it was routine work in addition to producing products for COVID, much pressure, tiredness, physical exhaustion” MGF_BS2. This new work dynamics led to a feeling of pressure to increase efficacy and performance, “there was so much administrative pressure to establish measures quickly, but the shortage of personnel overloaded the team” MGF_CI1.

Conversely, teleworking for administrative tasks facilitated the performance of work, despite increasing workload, because “one works more virtually than face-to-face, losing boundaries, without knowing when to stop” MGF_BS2, “it’s been forgotten that we’re also ordinary common human beings, we get tired and suffer like everyone else” MGF_HMI1, with combined loads: “Leaving work extremely tired, arriving home even more tired, and then doing the housework; there was a moment when I rebelled against it and said that’s enough, I can’t keep doing so many thing at the same time” HGF_GC2.

### FEELINGS

#### Fear of contaminating and being contaminated

This fear is now part of health staff’s everyday life “one could feel that the staff was tense and stressed, there was a fear of contagion” HGF_CJ4, “we were not familiar with the disease, we didn’t know what to do, and when we were told what to do, we didn’t know what to do it, we were very afraid” MGF_CP1. The fear of contaminating their loved ones led professionals to experience a double isolation, enforced separations, and detachment, COVID-19; “certainly went beyond everything [...] I had to leave my house, because of fear of contaminating my parents, here I am alone and I see them once a week” MGF_CP4, “I was afraid not only of being contaminated but also for my family, of contaminating my grandmother” HGF_CJ4; “the hardest thing of the pandemic was having to separate myself from the person who took care of me, helped me, did everything for me, absolutely everything, she was a 65-year old woman with heart conditions, and I had no choice but to separate myself from her” HFG_AC4.

“Fear never ended, fear that patients conveyed to their families was the fear experienced by the staff [...] I believe partly arising from lack of knowledge” HGF_JCG1. “Fear was also intensified by the excessive amount of information; we didn’t know what to do with it, it was possibly not true and nor that critical, but communication media and social networks has made it worse” MGF_BS3. Fear was contagious “we were afraid reflecting in Europe” [...] “we were afraid of reaching a similar stage, or even worse, because of the country’s social conditions” MGF_CP1.

#### Uncertainty, anguish, and powerlessness

Changes in health services, lack of knowledge, and feeling of wanting to achieve something impossible favored emotional distress, “we felt deep anguish for our economic viability, all consultations and diagnostic assistances decreased, [...] but at the same time we were taking a very large responsibility” MGF_IC1. Additionally, “when thinking about the future [...] would we be able to face the peak of the pandemic? [...]; I don’t know what will happen, nor when it will last,” all of this added to the “uncertainty of getting out the comfort zone to explore new paths” MGF_CP1.

#### Loneliness

“This is an absolute lonely disease, if people do not die from COVID, sadness and loneliness kill them” MGF_CI2. Loneliness is a desperate situation: “many patients are isolated and afraid, unable to see or talk to their families, because they did not have a cell phone. [...] Loneliness is not a good friend of death; die lonely, with no goodbyes, destabilizes, shakes up one’s feelings [...] made me lose control, I’m used to see my patients saying goodbye [...], I’ve never seen a happy ending with a patient with COVID, these are experiences totally contrary to those I wished they experienced. Providing a scarce consolation through a screen is seeing someone dying without doing much, this causes desperation and distress” HGF_AC2. Through the camera one feels more pain from the condition of the other who is near, whereas it becomes more difficult to the proper words and tone of voice for those who suffer at distance. MGF_IM2. However; “after several weeks without seeing the family, so much loneliness and pain, being able to show him to his family through a video call made both him and me very happy” MGF_PC2.

### SIDE EFFECTS

Pandemics has implied far-reaching changes for the health personnel, going from separation from their loved ones up to living stoically, “not being able to see and be with those we love, feeling that we should leave our personal life behind to help others” GFM_4_B; and understanding that there are other ways to live. “I acknowledge that you can live with fewer things than you think” GFM_4_CI. From another point of view, “this is a loss, seen from the collective point of view” MGF_HM2.

The quality of health care was also affected, “the number of patients’ falls increased, as well as length of hospital stay, even with the occurrence of sentinel adverse events” HGF_JC3. Furthermore, “there were shortage of medicines and personal protective equipment,” and “since visits were suspended, the recovery process, specifically of psychiatric patients, has become longer, worsening their condition, due to the feeling of abandonment and loneliness” GFM_IM4. Conversely, wearing a mask affects the relationship with patients, hampering care provision; “it was hard for me to wear a mask, not the very act of wearing it, but in the practice with the patient, because I lose visibility, the patient also loses the possibility of postural echo, of preverbal reinforcement, and even of preverbal panel, because they don’t see me, but only my eyes, and through the glasses I have to wear. This entire situation has been completely difficult, because I need to see the patients, and they need to see me” GFM_IM4.

Moreover, professionals report a disruption of paradigms: “We were prepared to utilitarianism, in which we think more in the common good and consequences. We were trained to care for the individual, for the patient in front of us, not for the one yet to come or how many I can save. We’re not prepared to establish a balance between being able, wanting, and having to, even less in a situation like that” HGF_JC1.

### COPING STRATEGIES

#### Grief

“There was a transition that I don’t know how to describe (denial of reality, adjustment to death?), the reality was so overwhelming that there is no way to avoid it, and I believe we started to speak the language of seeing and silencing; just accepting what happens and being there” HGF_CAG2. In this transition, “we face a reality that we can’t change, there’s no treatment, while there’s no vaccine or something new, we’ll simply do as much we can, I don’t know if I can call it acceptance. This was a process of disease and grief. This is how I feel...” HGF_CAG1.

#### From standardization to flexibility

“When the use of personal protective equipment was standardized and protocols were socialized, there was a slight improvement in the situation; however, I believe that it is still very hard dealing with families, visitors, and, particularly, with the topic of end of life. We have made exceptions to protocols and rules: letting an older adult come in to see a patient at the end of her life, because he was the only relative who did not have the technology to make a video call” HGF_JCG1. There, “it is necessary to confront the rule with the human aspect, I can’t be so strict, because I have to consider each individual situation, each situation is different” MGF_AL2, and “we constantly asked ourselves: “are we doing the right thing?” and we often reformulated what we were doing” HGF_JC3. Hospitals have also changed: “mi clinic has changed [...] the number of ICU beds has suddenly increased from 60 to 140” HGF_CAG1.

#### Compassion/empathy

“I had the experience of having my grandmother hospitalized due to COVID, I was a companion, a relative, and the chief of those who were treating her, it was very hard. Experiencing this even closely with a relative or with friends from work allowed me to understand others better, being more flexible, respectful, and work better as a team” HGF_JC3, furthermore, “I have found that one can hug people from distance with the power of words; so, although we can’t hug them, we can help them” MGF_CP1.

#### Asking for help and assertive communication

“There was a high demand of psychiatrists from the health care staff; which, more than an option, became an obligation, a moral duty. They saw psychiatrists performing empathic listening and we just validated other’s feelings, recognizing them, normalizing them somehow in the middle of an abnormal situation, to reduce anxiety” MGF_HMI1. “It was necessary to build a communication bridge between all people, patients, families, and coworkers, aiming to reach an understanding that creates consensuses” HGF_JC3.

### CARE AND SELF-CARE

Professionals are aware that they put their lives in danger by caring for others, “all of us were trained to practice detachment, we’re aware of the risk to which we’re exposed, we often help without thinking about our own risk, but this is how we were trained, this is how we are, this is what we do, caring for the ill implies a risk” MGF_AL3. However, “Much empathy and assertiveness are required to tell others: if you take care there will two less hands and one more patient [HGF_JC2]. “Here is not the place to take heroic measures, I have to think first in protecting myself before caring for a patient, so, if it necessary to perform an urgent intubation, we must think in the issue of protection first, but it’s hard when the time comes” MGF_AL3. “We’re always ready to serve, here we don’t make toy cars or plastic boxes, here we work to save lives” MGF_BS3. Finally, “it is important to acknowledge the work of health professionals, with a fair payment (social and economic), which can help mitigate stress” HGF_CG3.

### RESILIENCE

“In very hard situations, resilience appears as a way to face them HFG_AC4, “I learned to exercise daily, before going to the office” MGF_BS4, “everything changed, a change of paradigm implies a mobilization of emotions, that is, a different way of doing things, which doesn’t imply that this way is necessarily worse or better” GFM_IM4. “The key word has been reinvention, understanding that we used to do had worked before, but it was not the only to see and do things, to care for themselves and to care for patients. HGF_CG3 “I found out how I can innovate, change, and adapt, to see the bright side despite hardships. When the changes take effect, they motivate us not to lose heart and to go on” HGF_JC3; “I believe this type of circumstance take out the best of people” HGF_CG2.

### FAMILY RELATIONSHIPS

“Pandemic has caused important changes, such as consolidating the bond with my husband” MGF_4IM, “due to the strengthening of our relationship as a couple, we’re able to take a step ahead and keep strengthening our bonds, going back to the basics, to the simple” HGF_4_CJ. Although “spending so many hours with my family, respecting other’s tastes and everything, it’s not that easy, but it’s possible” MGF_4_CI. “I learned to prioritize what is most important, my family. Life is today, not tomorrow, I don’t know what will happen within a year, a daily hug is surely necessary, and I need it so much when I was alone. Being healthy and being close to our loved ones is our greatest wealth, love is surely the best answer to any situation we have to face” MGF_4_C.

## DISCUSSION

The experiences reported by the professionals participating in this study in relation to changes in their work dynamics, implying in increased workload, reduced health human resources, and deterioration of working conditions, have also been described by the International Labor Organization (ILO)^14^ and by other studies both in nursing professionals^15^ and in the health field,^16^ which found greater overload and more adverse conditions in low-income countries.^17^

COVID-19 pandemic has represented what could be considered a war scenario^10^ or similar to a natural disaster, promoting feelings like those reported by participants in this study, which in part emerge as a response to a stressful event that is outside the range of the usual human experience. Health care during large scale disasters or epidemics has been associated with a significant increase in the occurrence of mental health disorders both in immediate and long term periods, leading to increased rates of posttraumatic stress disorder, depression and substance abuse disorders, associated with increased workload and compassion fatigue,^18^ feelings that were partly evidenced among professionals who participated in this study.

The fear of contaminating and being contaminated “is a generalized fear” HGF_CAG1, anguish and uncertainty are closely related to the social, family and individual contexts; in a global context like that of the pandemic, they even go beyond the emotional domain, until the development of somatic symptoms.^19^ Therefore, those who have experienced and treat the disease can acknowledge that, as shown in this study, “COVID-19 is an absolutely lonely disease” MGF_CI2, social isolation makes loneliness chronic and intensifies it, splitting family unity in the health-disease process and affecting patients, health care professionals, and the general population^20^; in a hyperconnected world, the bonds that remain are the virtual ones, and actual bonds become more distant and weak amidst the pandemic.^21^

Other consequences associated with COVID-19 and workers’ professional wellbeing, as evidenced in the present study, are related to reduced quality of health care, leading to an increased number of adverse events,^22^ hospital infections, care errors, decreased productivity,^23^ high health care costs and increased demands,^24^ in addition to dissatisfaction of patients, families, and professionals due to changes in health care.^25^

As reported by Zhai & Du,^26^ multiple losses (relationships, freedom, job, health, among others) are detrimental to physical and mental health, posing civilians and health personnel at risk for grief overload. Society and, especially, health care professionals experience a grief due to all these losses, being heroes and victims at the same time. Similar to this adaptive grief process, the reports of the professionals in this study also portray the stages described by Kübler-Ross,^27^ related to denial, anger, bargaining, depression, and acceptance; “feeling of powerlessness and not being able to do more; “we questioned ourselves all the time ... I felt that I couldn’t make it, that this situation was overwhelming” HGF_CAG2.

According to Heath et al.,^28^ the need to develop resilience has gained impetus in recent times, consisting of the ability to resist disruption of normal functioning in the face of a distressing event, by anticipation or preparation. Other authors^29^ pointed that resilience is an important factor that differentiates physicians with and without burnout syndrome. However, it is not an individual’s innate capacity, its configuration is closely linked to psychosocial aspects. Conversely, it is necessary to acknowledge and give visibility to the situation surrounding the pandemic in all social domains, acknowledging other’s suffering and the stigmatization experienced by patients and health care professionals,^30^ as well as the work of these professionals. Acknowledging is the first step to solve a crisis,^31^ in addition to establishing strategies that facilitate care to patients, the general community, and health care professionals.

## CONCLUSIONS

In conclusion, isolation during the pandemic, from March to September 2020, increased health care professionals’ workload, causing reassignment of roles. They also experienced fear, uncertainty, fear due to lack of knowledge, affecting health care, with increases in length of hospitalization and number of adverse events, and making health care professionals lose their sensibility and become adjusted to death. Due to fear of being contaminated and contaminated, they experienced a double isolation, with anguish and uncertainty; thus, now they give priority to what is most important in their lives: health and love.
